# Clinical, laboratory data and outcomes of 17 Iranian citrullinemia type 1 patients: Identification of five novel *ASS1* gene mutations

**DOI:** 10.1002/jmd2.12277

**Published:** 2022-03-09

**Authors:** Shirin Moarefian, Mahdi Zamani, Ali Rahmanifar, Babak Behnam, Talieh Zaman

**Affiliations:** ^1^ Department of Neurogenetics, Iranian Center of Neurological Research (ICNR) Tehran University of Medical Sciences Tehran Iran; ^2^ Clinical and Research Unit Iranian National Society for the Study of Inborn Errors of Metabolism Tehran Iran; ^3^ Department of Medical Genetics, School of Medicine Tehran University of Medical Sciences Tehran Iran; ^4^ Department of Medical Genetics and Molecular Biology Iran University of Medical Sciences Tehran Iran; ^5^ Metabolic Unit of the Children's Medical Center, School of Medicine Tehran University of Medical Science Tehran Iran; ^6^ Present address: Amarex Clinical Research, Department of Regulatory Affairs, Germantown Maryland USA

**Keywords:** *ASS1*, citrullinemia type 1, clinical, Iran, novel mutation, outcome

## Abstract

Citrullinemia type 1 is an autosomal recessive metabolic disease caused by *ASS1* gene mutations encoding argininosuccinic acid synthetase enzyme which is within the pathway of arginine and nitric oxide biosynthesis. Disease confirmation was done by *ASS1* gene mutation analysis using next‐generation sequencing, DNA Sanger sequencing. The study group was 17 citrullinemia type 1 patients from 10 unrelated families referred to Iranian National Society for Study on Inborn Errors of Metabolism's clinic between 2008 and 2020. Clinical, laboratory, and molecular data were retrospectively evaluated. Eleven different *ASS1* gene mutations were detected in 13 (76%) of 17 neonatal, three (18%) of 17 late infantile, and one (6%) of 17 asymptomatic patients. Severe developmental delay and intractable seizures despite metabolic control was outcome of neonatal form survivor. Two late infantile form patients live metabolically controlled with quite normal performance. DNA mutations are as follows: seven missense, one nonsense, and two insertion/deletion mutations in 12, two, and three patients, respectively. Five novel mutations were detected including a homozygous GG deletion in exon 12 (c.790_791delGG;p.Gly264Profs*3) and a homozygous mutation in exon 7 (c.440C>T; p.Met147Thr), both causing infantile (late onset) form; a homozygous mutation in exon 6 (c.1130T>C; p.Met376Thr) causing neonatal form; two compound heterozygote mutations in exon 14 (c.1167_1168insC:p.Gly390Argfs*22& c.1186T>A; p.Ser396Thr) causing asymptomatic form. Five (38%) patients with classic neonatal form had mutation in exon 14 of *ASS1* (c.1168G>A; p.Gly390Arg). Classic neonatal was the most common form of disease in Iranian‐studied patients and homozygote c.1168G>A was the most frequent *ASS1* gene mutation. Global neonatal screening for citrullinemia type 1 in Iran is recommended and certain mutations can be used for screening severe form in this population.

**Synopsis**

Citrullinemia type 1 is a quite difficult curable disease and this article suggests that prenatal diagnosis, affected newborn birth prevention, and newborn metabolic screening are necessary in Iranian population. Carrier detection in consanguineous Iranian couples for severe form can be done using c.1168G>A mutation.

## 1 INTRODUCTION

Citrullinemia type 1 (CTLN1), or argininosuccinate synthetase (ASS) deficiency, is a rare autosomal recessive metabolic disorder caused by mutations in the *ASS1* gene.^1^ It is a urea cycle disorder that estimated incidence is one in 44 300–250 000, based on the literature on newborn screening using mass spectrometry.^2^, ^3^, ^4^ CTLN1 shows heterogeneous clinical manifestations. The classic neonatal‐onset form involves hyperammonemic encephalopathy with lethargy, failure to thrive, seizures, coma, and death early in life. Milder phenotypes (late onset) show neurologic impairment, somnolence, and chronic intermittent hyperammonemia during infancy, childhood, and adulthood. Some individuals (biochemical phenotype) have an asymptomatic clinical course.^1^, ^5^, ^6^ To date, at least 137 mutations in *ASS1*, including gross deletions/duplications, have been associated with CTLN1.^3^, ^6^, ^7^ Biochemically, ASS deficiency is characterized by hyperammonemia, hypercitrullinemia, and orotic aciduria.^1^

Even in countries where CTLN1 is included in newborn screening, affected babies may become symptomatic (lethargy, coma) before screening results are available.^2^

In many developing countries, ammonia measurement is not widely available. Also, many primary care physicians are not aware of disease symptoms which could lead to late diagnosis.^2^ Almost all of the CTLN1 patients in this study presented with somnolence, poor feeding, vomiting, coma, and seizure. An accurate genetic diagnosis can be helpful not only for better disease management but also for genetic counseling for subsequent pregnancies and carrier detection.^8^, ^9^ In Iran, comprehensive neonatal metabolic screening is not broadly available. Also, there is no available report about *ASS1* gene mutations in Iran region. As the Iranian population is a mix of several ethnicities with considerable consanguinity, there is a high demand for genetic diagnosis of *ASS1* gene mutation for early detection and timely treatment; prenatal diagnosis (PND) is also necessary. Here we present our experience in diagnosis, genetic testing, and outcome after diagnosis of CTLN1.

## 2 METHODS

This is a descriptive retrospective study on 17 patients affected by CTLN1 who were treated at the time of diagnosis and their outcome and genetics are reported and analyzed compared with literature. The data were collected from patients referred to Iranian National Society for the Study on Inborn Errors of Metabolism's clinic. Seventeen patients' data out of 10 unrelated families were analyzed during follow‐up from 2008 to 2020.

### 2.1 Study design

Seventeen CTLN1 patients from 10 unrelated families were enrolled in the study. The Iranian National Society for Study on Inborn Errors of Metabolism in Tehran is an institution that accepts patients from every province of Iran. The current study included patient reports between 2008 and 2020. Diagnosis was based on clinical symptoms and laboratory confirmation, elevated plasma citrulline levels determined by mass spectrometry upon newborn screening or by high performance liquid chromatography later, hyperammonemia determined by an enzymatic spectrophotometric assay (Randox Ammonium Kit, Randox Laboratories LTD, UK), and orotic aciduria determined by thin layer chromatography (normally not detected). Standard treatment with ammonia scavengers, peritoneal dialysis, exchange transfusion (if dialysis was not available), l‐arginine, l‐carnitine, a low‐protein diet, and special urea cycle defect formulas was carried out after diagnosis. Then, age of onset, clinical presentation, plasma citrulline, glutamine and ammonia levels, clinical outcome, family history, and molecular genetic analysis were retrospectively evaluated.

### 2.2 Molecular genetic analysis

Based on clinical and biochemical findings, the coding region and 5′‐, 3′‐untranslated regions of the *ASS1* gene (ENST00000352480; NM_054012) were analyzed by polymerase chain reaction or next‐generation sequencing and confirmation of disease was made via molecular genetic analysis. All detected variants in patients and their parents were validated utilizing Sanger sequencing at the diagnostic genetic laboratories of Iran and Tehran Universities of Medical Sciences. If the patient's DNA was not available because of early death, parents' DNA were analyzed. Mutation analysis was done after parental consent was obtained. For families who planned for a healthy child, PND was performed by amniocentesis at 13–14 weeks' gestation. Mutation analysis was carried out in fetal DNA using Sanger sequencing to determine whether a fetus is affected with previously identified mutations. The primers and sequences are available upon request.

## 3 RESULTS

**FIGURE 1:**
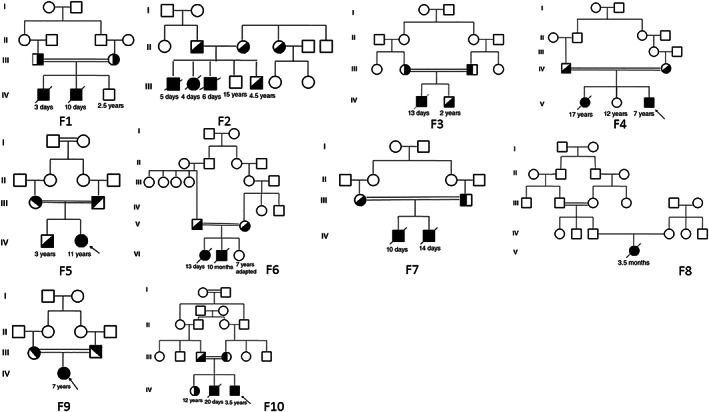
The pedigrees of families affected by citrullinemia type 1 (CTLN1)

Seventeen patients (seven female; 10 male) affected by CTLN1 from 10 unrelated families enrolled in this clinical, genetic study. Eight patients had consanguineous parents (Figure 1). Figure 1 indicates pedigrees of affected CTLN1 families. Ten different mutations (consisting of nine homozygous and one compound heterozygous mutation), including five novel variations, were detected in this cohort's DNA. The mutations included seven missense, one nonsense, and two insertion/deletion mutations in 12, two, and three patients, respectively .Table 1 shows demographic and clinical (presentation, outcome) phenotype of CTLN1 patients. The seven missense mutations included: (1) patients number 1 through 5: c.1168G>A and p.Gly390Arg; (2) patient number 6: c.470G>A and p.Arg157His; (3) patient number 9 (compound heterozygote): c.1186T>A and p.Ser395Thr; (4) patients number 10 through 11: c.1130T>C and p.Met376Thr; (5) patients number 12 through 13: c.350G>A and p.Gly117Asp; (6) patient number 14: c.1022T>C and p.Ser341Phe; and (7) patient number 15: c.440T>C and p.Met147Thr. The only detected nonsense mutation was c.835C>T; p. Arg279fs* (in P_16_–P_17_). The two insertion/deletion mutations were: c.790_791delGG; p. Gly264Profs*3 (in P_7_–P_8_) and c.1167_1168insC; p.Gly390Argfs*22 (compound heterozygote in P_9_). Table 2 shows biochemical and molecular characteristics of *ASS1* variants in CTLN1 patients. All of the above‐mentioned variations were predicted to be deleterious mutations via testing by Mutation Taster.^13^ Five of the variations were previously reported as disease causing in CTLN1 patients.

**TABLE 1:** Demographic, clinical phenotype (presentation, outcome) of Citrullinemia type 1 patients

Clinical phenotype									
F#	Proband	Gender	Parental consanguinity	Age at diagnosis	Age at last follow‐up	Clinical phenotype	Number of crisis	Outcome	Treatment
F_1_	P_1_	M	Y	3 days	3 days	Neonatal	1	Deceased	Peritoneal dialysis + standard treatment
	P_2_	M	Y	5 days	10 days	Neonatal	2	Deceased	Hemodialysis + standard treatment
F_2_	P_3_	M	N	5 days	5 days	Neonatal	1	Deceased	Peritoneal dialysis + standard treatment
	P_4_	F	N	2 days	4 days	Neonatal	1	Deceased	Peritoneal dialysis + standard treatment
	P_5_	M	N	2 days	6 days	Neonatal	1	Deceased	Peritoneal dialysis + standard treatment
F_3_	P_6_	M	Y	3 days	13 days	Neonatal	1	Deceased	Peritoneal dialysis + standard treatment
F_4_	P_7_	F	Y	9 months	17 years	Late onset	5	Intellectually disable + seizure	Without treatment
	P_8_	M	Y	8 months	7 years	Late onset	3	Intellectually normal + seizure	Standard treatment
F_5_	P_9_	F	Y	8 years	11 years	Asymptomatic	0	Short stature	Without treatment
F_6_	P_10_	F	Y	3 days	9 days	Neonatal	2	Deceased	Hemodialysis + standard treatment
	P_11_	M	Y	5 days	10 months	Neonatal	3	Deceased	Hemodialysis + standard treatment
F_7_	P_12_	F	Y	3 days	10 days	Neonatal	1	Deceased	Without treatment
	P_13_	M	Y	4 days	14 days	Neonatal	1	Deceased	Peritoneal dialysis + standard treatment
F_8_	P_14_	F	N	3 days	3.5 months	Neonatal	4	Deceased	Peritoneal dialysis + standard treatment
F_9_	P_15_	F	Y	9 months	7 years	Late onset	3	Intellectually normal	Standard treatment
F_10_	P_16_	M	Y	3 days	20 days	Neonatal	1	Deceased	Peritoneal dialysis + standard treatment
	P_17_	M	Y	3 days	3.5 years	Neonatal	5	Intellectually disable + seizure	Hemodialysis + standard treatment

**TABLE 2:** Biochemical phenotypes and molecular characteristics of *ASS1* variants in citrullinemia type 1 patients

Biochemical phenotype						Genotype characteristics					
F#	Proband	Amonia^a^	Citrulline^b^	Glutamine^c^	Urine orotic acid^d^	Location	Nucleotide change	Predicted effect	Function	Frequency	Reference
F_1_	P_1_	470 ± 275	1800 ± 130	1500 ± 150	+	Exon 14	c.1168G>A	p.Gly 390 Arg	Missense, Hom.^e^	5/17	Ref. 1
	P_2_	500 ± 250	1652 ± 148	1600 ± 120	+						
F_2_	P_3_	340 ± 100	2046 ± 200	790 ± 100	+	Exon 14	c.1168G>A	p.Gly 390 Arg	Missense, Hom.	5/17	Ref. 1
	P_4_	540 ± 200	2450 ± 250	890 ± 150	+						
	P_5_	500 ± 100	1489 ± 150	900 ± 100	+						
F_3_	P_6_	300 ± 150	777 ± 100	800 ± 200	+	Exon 7	c.470G>A	p.Arg157His	Missense, Hom.	1/17	Ref. 10
F_4_	P_7_	NA	NA	NA	NA	Exon 12	gg deletion:c.790–791	p.Gly264Profs*3	Deletion, Hom.	2/17	Novel
	P_8_	150 ± 50	1500 ± 300	700 ± 100	+						
F_5_	P_9_	50 ± 10	1298 ± 100	500 ± 100	+	Exon 15	c.1168inserc&c.1186T>A	p.Gly390Argfs*22&p.Ser396Thr	Insertion, Com.^f^ Het.^g^	1/17	Novel
F_6_	P_10_	370 ± 150	2927 ± 200	1700 ± 100	+	Exon 6	c.1130T>C	p.Met376Thr	Missense, Hom.	2/17	Novel
	P_11_	730 ± 200	2469 ± 100	1780 ± 100	+						
F_7_	P_12_	NA	NA	NA	NA	Exon 5	c.350G>A	p.Gly117Asp	Missense, Hom.	2/17	Ref. 10
	P_13_	700 ± 200	758 ± 100	900 ± 200	+						
F_8_	P_14_	200 ± 20	1536 ± 200	900 ± 100	+	Exon 14	c.1022T>C	p.Ser341Phe	Missense, Hom.	1/17	Refs 3, 11
F_9_	P_15_	100 ± 50	3515 ± 120	1100 ± 200	+	Exon 7	c.440T>C	p.Met147Thr	Missense, Hom.	1/17	Novel
F_10_	P_16_	NA	NA	NA	NA	Exon 12	c.835C>T	p.Arg279fs*	Nonsense, Hom.	2/17	Ref. 12
	P_17_	705 ± 25	1154 ± 100	950 ± 50	+						

^a^ Normal <47 μmol/L.

^b^ Normal <45 μmol/L.

^c^ Normal <600 μmol/L.

^d^ Normally not detected.

^e^ Homozygote.

^f^ Compound.

^g^ Heterozygote.

Among five novel variations, two were found in only asymptomatic patient (P_9_ in F_5_) who was heterozygous compound for a variant located in exon 14 of *ASS1* (c.1167_1168insC: p.Gly390Argfs*22 and c.1186T>A; p.Ser396Thr). Two other novel mutations were found in the three late‐onset CTLN1 patients (P_7_, P_8_, and P_15_) from two unrelated families (F_4_ and F_9_), located in exon 12 (c.790_791delGG; p.Gly264Profs*3) and exon 7 (c.440T>C; p.Met147Thr) of the *ASS1* gene. Also an additional *ASS1* novel variant in two classic neonatal CTLN1 patients (P_10_ and P_11)_ from one family (F_6_) was homozygous located in exon 6 (c.1130T>C; p.Met376Thr). Among 10 of studied families, seven presented during the neonatal period (70%) and two (29%) were homozygous for c.1168G>A; p.Gly390Arg located in exon 14 of *ASS1*. PND was performed for three families (F_1_, F_2_, and F_3_) via amniocentesis at 13 weeks' gestation and none were affected.

All patients were born to uncomplicated pregnancies and deliveries. Sixteen patients were born by elective cesarean section. The mean birth weight was 3150 g (range: 2600–4250 g). Thirteen (76%) of 17 patients had classic neonatal CTLN1 with a mean onset of 3.2 days (range: 2–5 days) and mean ammonia, citrulline, and glutamine levels of 486 ± 175 μmol/L (range: 200–705) and 1732 ± 699 μmol/L (range: 758–2927), 1030 ± 285 μmol/L (700–1600), respectively. Ten (76%) of the 13 died at a mean age of 9.8 days (range: 3–20 days) despite peritoneal/hemodialysis and standard pharmacological treatment (ammonia scavengers, l‐ carnitine, l‐arginine, and protein restriction). The remaining 3 (P_11_, P_14_, and P_17_) were discharged home at a mean age of 24 days (range: 11–38 days). P_11_ had normal growth and development with standard treatment until 10 months of age but succumbed during a severe hyperammonemic attack due to respiratory infection. P_14_ lived until 3.5 months but had a severe delay in growth and development and frequent readmissions to the hospital for peritoneal dialysis. P_17_ was discharged at 11 days after two exchange transfusions and developed fairly well until 11 months when he had a severe seizure and began to regress. Currently at 4 years of age, he is unable to walk or speak and has refractory seizures despite metabolic control by standard treatment. Three out of 17 patients (17%; P_7_, P_8_, and P_15_) were admitted with late‐onset CTLN1 and mean age at onset of 8 months (range: 8–9 months). Their mean ammonia, citrulline, and glutamine levels were 125 ± 35 μmol/L (range: 75–160), 2507 ± 1427 μmol/L (range: 1015–3515), and 900 ± 282 μmol/L (range: 700–1100), respectively (Table 1). P_7_ presented at age 8 months with vomiting and seizures but was undiagnosed and untreated until she died at 17 years of age, bedridden with refractory seizures because of late diagnosis. P_8_, her brother, is now an 8‐year‐old boy who presented with vomiting, seizures, and developmental delay at 9 months of age when standard treatment for CTLN1 was started. He does fairly well at school. He is currently metabolically controlled but has episodes of intermittent hyperammonemia and seizure is controlled. Enzyme assessment of this patient from peripheral blood showed 50% enzymatic activity of argininosuccinate synthase: 0.5 nmol/kg/mg protein (range: 0.8–8) before genetic test result was available, which confirmed the diagnosis. P_15_ presented at 9 months of age with lethargy, vomiting, and slight motor delay, when standard treatment was initiated. Currently, she is 7 years old and she is free of seizures and well‐controlled mentally, physically, and metabolically with good school performance. P_9_, the only asymptomatic patient, has been followed since she was 2.5 years old when she was referred to the clinic for poor weight gain and no other symptoms. During the follow‐up for her constitutional growth delay, the high citrulline levels were observed. Laboratory tests were repeated yearly and she has remained developmentally normal without any clinical symptoms. However, standard therapy (l‐carnitine, l‐arginine, low‐protein diet) neither change her growth rate nor her biochemical abnormality. Therefore, treatment and protein restriction were discontinued at 11 years of age and she is now under annual follow‐up appointments. She is currently symptom free yet, still biochemically abnormal. Advice was given to the patient/family to follow her symptoms closely during pregnancy and major metabolic stresses.

## 4 DISCUSSION

ASS is a rate‐limiting enzyme in the urea cycle coded by the *ASS1* gene located on chromosome 9q34.1 which contains 16 exons.^1^ Classic CTLN1 require long‐term surveillance with diligent nutritional and pharmacological management associated with high morbidity and mortality despite treatment.^14^ Today CTLN1 is included in some newborn screening programs in which elevated citrulline is detected in dried blood spots by tandem mass spectrometry.^2^ Consanguinity and close community marriages are important factors leading to increased occurrence of recessive disorders.^10^ PND of risky pregnancies requires prior identification of disease causing mutation within the family.^5^ There are two main reasons for PND of urea cycle disorders: (1) to enable parents to terminate affected pregnancies or (2) to allow precautions to be taken immediately after birth.^5^

There is high rate of consanguinity marriages in Iranian population and newborn screening is not performed globally for CTLN1. Also, ammonia measurement is not available in all pediatric care centers. In this study, we described 17 patients who were/are affected by CTLN1, including 13 neonatal, three late onset, and one asymptomatic patient. This study was similar to two previous studies on Indian and Korean CTLN1 patients in terms of showing a high prevalence of neonatal presentation.^10^, ^15^ Eleven different *ASS1* variations, including five novel and six previously reported mutations,^1^, ^3^, ^5^, ^11^, ^12^, ^14^, ^16^, ^17^, ^18^, ^19^, ^20^, ^21^ were ascertained in our patients. Although biochemical and enzymatic studies using tissue and fibroblasts are important for the diagnosis of CTLN1,^9^ the molecular genetic investigation of the *ASS1* gene can help establish a genotype–phenotype correlation, understand phenotypic variability, and comment on prognosis.^8^, ^12^, ^17^, ^19^ The enzymatic activity of fibroblasts may be normal in children with citrullinemia or, conversely, have no enzymatic activity in asymptomatic patients; thus, sequencing of the *ASS1* gene is recommended to confirm diagnosis and predict the prognosis of citrullinemia patients.^3^, ^11^, ^16^ It also allows PND for future pregnancies and carrier detection within affected families for genetic counseling.^11^, ^20^

In this study, the majority of variations occurred in exon 14 (47%) in four out of the 10 independent families (40%). This was followed by variations in exons 11 and 6 as the second and third most frequent mutations, respectively. Exons 14, 11, and 6 of the *ASS1* gene are suggested to be hot spots for tier 1 screening of patients with CTLN1 in Iran. This is compatible with other studies showing that most mutations of the *ASS1* gene are distributed throughout the gene, except for exons 5, 12, 13, and 14.^5^, ^12^, ^17^ The majority of patients in our study (94%) were homozygous for *ASS1* gene mutations; the only one patient (P_9_) with compound heterozygote mutations (c.1167_1168insC and c.1186T>A) was clinically asymptomatic but biochemically affected. Interestingly, this asymptomatic case (P_9_ in F_5_), which also showed compound heterozygote for variants in exon 14 of the *ASS1* gene, is also the product of consanguinity. This pattern was compatible with previous studies in which mild/asymptomatic phenotypes probably had residual enzyme activity.^11^, ^12^, ^17^, ^18^ In another study, a woman with *ASS1* compound heterozygous mutations who had a mild phenotype, presented with severe symptoms during pregnancy and postpartum.^17^, ^19^ Most of our patients showed missense homozygote mutations of the *ASS1* gene, with 94% of them being affected of a severe neonatal form of CTLN1. Therefore, in this study, similar to some previous studies,^3^, ^10^ missense mutations were accompanied by early‐onset severe clinical courses. In this study, only two patients (P_11_ and P_15_) out of seven patients carrying missense mutations of *ASS1* gene were neurologically normal under treatment. Gao et al. showed that the phenotype of certain missense mutations is mostly late onset/mild.^12^ The majority of described *ASS1* mutations are missense mutations; however, patients show highly variable clinical courses.^18^ It can be concluded from previous studies^1^, ^17^, ^18^ and current study that a reliable prognostic factor for the course of the disease is still to be found and that genotype–phenotype correlation is not very strong. Future studies with genotype, phenotype, and enzyme measurement are recommended to find a correlation. However, our small study on 17 Iranian CTLN1 patients suggests that some missense *ASS1* mutation, if present in a compound‐heterozygote state may have a mild or even asymptomatic course of the disease.

A paucity of data on genotype–phenotype correlations and the various genotype combinations also cause confusion in the prediction of the clinical course of the disease according to genotype.^1^, ^3^, ^12^, ^17^ Many (38.5%) of our neonatal classic CTLN1 patients (P_1_–P_5_) were homozygous for a common mutation of *ASS1* (c.1168G>A; p.Gly390Arg), which caused the most fulminant disease course, leading to liver failure and early neonatal death despite treatment. This is consistent with the literature.^1^, ^3^, ^5^, ^7^, ^10^, ^11^, ^12^, ^14^, ^16^, ^17^, ^18^, ^19^, ^20^, ^21^ Only one of our classic neonatal CTLN1 patients (P_17_) is currently alive. He is a 3‐year‐old boy who cannot walk independently and has intractable seizures and speech delay despite ammonia control. This confirms the poor prognosis of the classic neonatal form of the disease as expressed in previous studies.^3^, ^10^, ^14^, ^15^

Mean citrulline levels were 1732 ± 699, 2507 ± 1427, and 1298 ± 100 μmol/L in our neonatal, late‐onset, and asymptomatic CTLN1 patients, respectively (range: 758–3515) (Table 1). The initial citrulline concentrations were less than 1000 μmol/L in some of the severe neonatal patients and more than 1000 μmol/L in the late‐onset and asymptomatic CTLN1 patients in our study group. However, it is still unclear whether the level of citrulline alone enables classification of patients.^3^, ^17^, ^18^, ^20^ During follow‐up examinations, the late‐onset and asymptomatic CTLN1 patients showed lower ammonia levels during metabolic stress compared to neonatal classic patients (<50 μmol/L vs. >100 μmol/L). Although the mean citrulline levels were high (>1000 μmol/L) in all patients who had attacks of metabolic decompensating, the late‐onset and asymptomatic patients in our study group had better outcome than the neonatal classic patients.

This is only an experience observed in our limited numbered study group. This fact that isolated episodes of hyperammonemia are not as deleterious as cumulative exposure to moderately high levels of ammonia or citrulline^21^ may be better to be investigated separately in different types of CTLN1 patients.

The activity of ASS is likely a useful and essential tool in predicting long‐term outcome and for interpreting functional effects of sequence variants.^3^, ^9^, ^16^ A better understanding of each mutation's effect would enable prediction of metabolic decompensation and lead to better management to prevent neurologic sequel.^2^

Liver transplantation remains the only curative treatment for CTLN1; however, due to limited availability of donor livers, substitute therapies are currently under study.^22^

Gene therapy has been suggested, but there is no successful treatment reported.^23^ Human hepatocyte transplantation as an alternative to liver transplantation has recently finished Phase II clinical trials.^23^ Unfortunately liver transplantation is not performed for inborn errors of metabolism in Iran as well as hepatocyte transplantation, and gene therapy. These are open fields for novel treatments in this region.

Finally, PND was done successfully in three of our 10 affected families, and no mutation was observed. PND is recommended for families affected by CTLN1 because our study shows that the majority of mutations do not respond well to treatment even if started early in the disease course.

This study was done on a small group of patients. A more extended study on more patients besides functional study for genotype phenotype correlation is suggested for future. It may be multicenter intercontinental to share clinical experiences.

## 5 CONCLUSION

Classic neonatal CTLN1 was the most common form of the disease in studied Iranian population and c.1168G>A was the most common *ASS1* mutation. Due to the poor prognosis of treated patients, prenatal diagnosis in affected families can prevent disease in subsequent pregnancies. Detection of genetic carriers in families affected by CTLN1 would be a useful means of diagnosis to prevent severe forms while managing the more curable forms at birth. As the majority of cases are the severe neonatal form in the studied population, global neonatal screening and specific gene mutation detection panels for severe forms is recommended for CTLN1.

## CONFLICT OF INTEREST

This research did not receive any specific grant from funding agencies in the public, commercial, or not‐for‐profit sectors. The authors declare no conflict of interest.

## AUTHOR CONTRIBUTIONS

Study design: Shirin Moarefian, Mahdi Zamani, Babak Behnam Data collection: Shirin Moarefian, Ali Rahmanifar, Talieh Zaman Statistical analysis: Shirin Moarefian Data interpretation: Shirin Moarefian, Babak Behnam Manuscript preparation: Shirin Moarefian, Babak Behnam, Talieh Zaman, Mahdi Zamani Literature search: Shirin Moarefian Funds collection: no funds

## ETHICS STATEMENT

All patient subjects gave their informed consent for inclusion before they participated in the study. The study was conducted in accordance with the Declaration of Helsinki, and the protocol was approved by the Ethics Committee of Tehran University of Medical Sciences (Project identification code: IR.TUMS.REC.1395.2327).

## Data Availability

The raw data supporting the conclusion of this article will be made available by the authors, without undue reservation.
